# Singapore’s Pandemic Preparedness: An Overview of the First Wave of COVID-19

**DOI:** 10.3390/ijerph18010252

**Published:** 2020-12-31

**Authors:** Jia Bin Tan, Matthew James Cook, Prishanee Logan, Liudmila Rozanova, Annelies Wilder-Smith

**Affiliations:** 1 Global Studies Institute, University of Geneva, 1205 Geneva, Switzerland; Matthew.Cook@etu.unige.ch (M.J.C.); Prishanee.Logan@etu.unige.ch (P.L.); Liudmila.Rozanova@unige.ch (L.R.); 2 Melbourne School of Population and Global Health, University of Melbourne, Bouverie St Carlton, VIC 3053, Australia; 3 Heidelberg Institute of Global Health, University of Heidelberg, 69120 Heidelberg, Germany; annelies.wilder-smith@lshtm.ac.uk; 4 Department of Disease Control, London School of Hygiene and Tropical Medicine, London WC1E 7HT, UK

**Keywords:** Singapore, case study, COVID-19, acute respiratory disease, epidemiology, non pharmaceutical intervention, country economy, social political disruption, media coverage, mathematical modelling, dormitories

## Abstract

A global response to the rapid spread of the 2019 novel coronavirus disease (COVID-19) is imperative in order to reduce mortality and morbidity as well as preventing a country’s health system from collapse. Singapore showed exceptional leadership in the containment of the spread of the virus, however through April 2020 the country experienced exponential growth in the number of infections, particularly migrant workers living in dormitories. The following historical case study provides an overview of Singapore’s country profile, their healthcare system and the country’s non pharmaceutical measures taken to mitigate and contain the spread of COVID-19 in the first few months of the pandemic. We explore the impact COVID-19 had on Singapore’s economy at that time and the implications of the resultant social and political disruptions. We conclude our study by using mathematical modelling to explore confirmed COVID-19 cases in Singapore’s local community and those living in dormitories and use this data to forecast the progression of the epidemic in Singapore given the non-pharmaceutical interventions in place at that time. Our results indicate the COVID-19 outbreak in Singapore increased 3-fold the initial doubling rate of 22.5 days in the first 2 months of the outbreak to 6.7 days in the 5th month; We note a faster doubling rate of 4.9 days for those living in dormitories compared to a doubling rate of 13.5 days for the rest of the community.

## 1. Introduction

The 2019 novel coronavirus disease (COVID-19) first emerged in the city of Wuhan, in China’s Hubei province in December 2019. Hypothesized to have surfaced after a human consumed an animal source sold at the Wuhan wet markets, COVID-19 became a global pandemic infecting over 3.3 million people across six continents and claimed the lives of almost 240,000 (*n* = 238,730) people in the first five months [1]. Although the properties of COVID-19 are still being researched, the World Health Organization (WHO) has recommended countries to adopt social distancing and quarantine measures in order to contain the spread of the virus (which spreads through respiratory droplets produced when an infected person coughs or sneezes) [1].

Singapore reported their first case of COVID-19 on 23 January 2020. Despite being a small island and situated in close proximity to the epicentre of the outbreak in Wuhan, China, Singapore gained international recognition in its efforts to mitigate the spread of the virus, keeping infectious rates low in the initial months of the outbreak. Neighbouring countries such as China, implemented extreme lockdown measures to stop the spread of the disease, however, Singapore managed to contain the outbreak of COVID-19 with minimum disruption to daily life in these first few months of the COVID-19 pandemic. This was largely due to their pandemic’s taskforce which was established soon after the outbreak of SARS (severe acute respiratory syndrome) in 2003. Since SARS, Singapore experienced outbreaks of H1N1 in 2009 and Zika thereafter in 2016, consequently the country has invested heavily in infectious disease prevention and preparation [2]. As soon as the COVID-19 outbreak was announced by WHO on 31 December 2019, Singapore acted quickly, implementing their risk mitigation measures as early as 2 January 2020. These measures included border control, mandatory 14-day quarantining for all returning residents [3], contact tracing and providing medical leave for mild cases allowing possible COVID-19 cases to self-isolate at home thus reducing community transmission [4]. For the month of February 2020, Singapore did not experience any exponential growth of infections of COVID-19. However, in early March 2020, Singapore saw a spike in the number of new confirmed COVID-19 cases believed to be caused by an influx of imported cases from European countries [5]. As of 1 May 2020, Singapore accounted for approximately 0.52% of global confirmed cases (*n* = 17,101) and 16 deaths [6].

The proactive measures Singapore took in the early months of the COVID-19 outbreak certainly had a profound impact on the containment of the virus. However, the change in trajectory through April 2020 of new COVID-19 infections raised concerns regarding Singapore’s mitigation strategies and the social, political, and economic impacts it had on the country. This historical case study from January 2020 through to May 2020 provides an overview of Singapore’s landscape and the initial impact COVID-19 has had on this small but highly efficient and populated country. We will review Singapore’s health care system, the country’s initial mitigation strategies to contain the spread of COVID-19 and the impact the disease has had on the country. We finally conclude our study by making possible outcomes of the pandemic using SEIR mathematical modelling [7]. However, it must be noted that the assumptions made in these models are based on a homogeneous population and assumes all individuals are affected equally. Although the model is limited it does give a general overview of how the pandemic can unfold. 

### 1.1. Country Profile

The Republic of Singapore is a small island city-state situated on the equator at the southern tip of the Malay peninsula, just north of Indonesia. It consists of one heavily urbanised main island of 724.2 km^2^ where most of the population live and about 54 small islands and islets which are virtually uninhabited [8].

Historically, the strategically located island was held by a succession of Malay kingdoms and sultanates before Samford Raffles established a British East India Company trading post on the island in 1819. Following an extensive period of contentious colonial rule and two tumultuous years as part of the newly emancipated Malaysia, Singapore emerged as an independent state in 1965 [9].

As a unitary parliamentary constitutional republic, the dominant People’s Action Party (PAP) has been the only ruling party to form government since independence. While Singapore has established effective rule-of-law, low corruption, and effective government there are concerns that civil liberties and parts of the political process are lacking [10].

Situated in the equatorial monsoon region of Southeast Asia, Singapore’s climate is tropical with uniformly warm weather and frequent rainfall [11]. With a population of 5.7 million, Singapore is one of the most densely populated nations in the world, at 7866 people per square kilometre.

Due to its colonial history, Singapore is a relatively multicultural society with people of Chinese descent making up 74.4% of the population, followed by Malays at 13.4% and Indians at 9%. 20% of the residents are aged below 20 years, 65% are aged between 20 to 64 years, and 15% are over 64 [12]. 

Singapore has four national languages enshrined in the constitution—Malay, Mandarin, Tamil, and English [13]. English is the language of instruction used in primary and secondary schools, and children also learn one of the other three official languages. This has resulted in a population who are almost universally bilingual [14].

With a highly developed market economy, globally rated as the most competitive by the World Economic Forum [15], and a per capita GDP of $SG 88,991, Singapore is one of the wealthiest countries in the world. As Singapore has limited natural resources the economy is predominantly service based (70%), with goods producing industries contributing the bulk of the remainder of the nominal GDP [16]. Singapore is a busy international financial and transport hub, home to the second largest port in Asia (after Shanghai) [17]. Over 19 million short-term visitors arrived in 2019 [18] and Singapore is ranked 6th on the Global Cities index identifying top performing and competitive cities [19].

Singapore has just over 1.4 million migrant or foreign workers. Of these 1 million workers hold “work permits” which are for low- to medium-skilled workers in the construction, manufacturing, marine shipyard, process (manufacturing of petroleum, petrochemicals, specialty chemicals and pharmaceutical products) and services sectors [20]. These workers have historically experienced worse health outcomes and are at a higher risk for infectious diseases than Singaporeans [21].

According to the Singapore Minister of Manpower, about 200,000 foreign workers were living in 43 large purpose-built dormitories during the pandemic [22]. These dormitories provide close living conditions with rooms housing between 12 and 20 workers using shared communal facilities, posing a considerable risk of communicable disease outbreaks [23].

In Singapore, all people covered by the Employment Act who have served their employer for at least three months are entitled to 14 days paid outpatient sick leave and 60 days hospitalization leave per year [24]. The Employment Act covers employees under a contract of service, including both local and foreign, full-time, part-time, and temporary workers. It does not cover seafarers, domestic workers, and civil servants, who have terms and conditions under their own individual employment contracts [25]. Even with widely available sick leave, presenteeism is an issue in Singapore. Prior to the COVID-19 pandemic outbreak Singaporeans would routinely present to work while suffering what they considered to be minor health conditions including headache / migraine, cough, and fever [26]. This presenteeism is primarily culturally related, and while the behaviour will be curbed during the overt pandemic, there are implications for planning the exit strategy from this crisis.

While the historically vibrant Singapore economy has delivered significant social benefits to its population, it faces a series of challenges in coming years. Domestically inequality remains a significant issue, softening growth failing to deliver significant welfare gains for average Singaporeans. This challenge will be exacerbated in coming years due to a population that is rapidly aging with a falling growth rate [27].

Singaporean society is collectivist with high power distance. Society exhibits quite weak uncertainty avoidance which reflects a tolerance in ambiguity and a desire to abide by rules due to the acceptance of unequal distribution of power rather than an innate need for structure, and people “… accept inequality but feel that the use of power should be moderated by a sense of obligation” [28].

### 1.2. Health Care System

Singaporeans have amongst the highest life expectancy at birth (84.8 years) and healthy life expectancy at birth (74.2 years) in the world. The largest contributors to Singapore’s disability-adjusted life year (DALY), the burden of early death and disability, are cardiovascular diseases, cancers, musculoskeletal disorders, and mental disorders. The bulk of Singapore’s DALYs are caused by non-communicable diseases, and there are key policy focuses on addressing these [29]. Prior to January 2020 one of the biggest challenges for the Singapore health system was managing sustainability and affordability into the future. Increased longevity and declining birth-rate mean that keeping citizens as active and healthy as possible is a critical component of managing healthcare costs [30].

As of 31 December 2018, there were 13,766 doctors and 42,125 nurses in Singapore, a care provider to population ratio of 1:410 and 1:134 respectively [31]. Singapore has 19 acute hospitals, with a combined total of 10,826 beds [32], giving a total of 1.8 beds per 1000 population which is well below the OECD average (4.7) and other developed Asia Pacific countries [33]. Given the generally good outcomes of the Singapore health care system, this figure may indicate a level of success of the local public health preventative care strategies.

The World Bank estimates that Singapore spends 4.44% of GDP on healthcare [34]. This figure is low, well below all OECD counties except Turkey. Despite this relatively modest amount of funding, the health system generally provides good outcomes to the population.

Singapore has established the financing of their healthcare system based on several key principles, namely that healthcare is an example of market failure (because producers can influence prices and consumers do not have the information required to seek the lowest price and maximise their utility [35]), that everyone should assume the key responsibility for their own health, and that savings should play a predominant role with insurance playing a secondary role.

In line with these principles, Singapore has developed several key initiatives to fund healthcare for citizens and residents, including:

- Medisave—a national medical savings scheme where all workers are required to save between 6.5%–9% of their monthly wage in a special savings account. These funds can be used to pay for medical expenses including hospitalisation and some approved outpatient treatments for the worker or an immediate family member.
- MediShield—a catastrophic medical insurance scheme which covers large hospitalisation bills resulting from serious injury or prolonged illness. Premiums are deducted from Singaporeans Medisave accounts and the scheme operates on an opt-out basis where newborns are automatically covered from their parents account.
- Medifund—a financial safety net established by the government to assist people needing financial support to pay hospital and healthcare bills.
- ElderShield—a severe disability insurance scheme providing basic financial protection to those who need long term care, especially during old age. It operates on an opt-out basis with Singaporeans automatically covered when they reach 40 years of age, with premiums being paid from their Medisave accounts [36].

Recently, a new ranking system designed to rate health system performance has been proposed. This ranking evaluates performance using nine indicators across three key domains—general performance, major clinical performance, and health system equity and sustainability. In this system each indicator is scored from 1 to 3, aggregated to a score out of 9 for each of the domains [37].

Each of these domains is graded A if the resulting score is 8 or 9, B if the score is from 5–7 and C if it is below 5. By this methodology Singapore has been rated ABB, which compares favourably with the USA, Russia, and China (ABC, BBC, BBC respectively) and comparable with the United Kingdom. The key domain which is most notably impacting on Singapore’s rating is Health System Equity and Sustainability, with less than 90% of the population having a Medisave account, and relatively low Government funding for health research.

### 1.3. Non-Pharmaceutical Interventions

Because of its location, climate, and status as a travel hub, Singapore has always been more vulnerable to communicable diseases. Both Enterovirus and Dengue are endemic in Singapore, and the country regularly sees imported cases of Chikungunya, Malaria, and Zika [38].

In 2003, SARS had a significant impact on Singapore, infecting 238 people of whom 33 ultimately died. Beyond the societal toll this pandemic had a significant effect on the economy, costing the state an estimated $US 4.9 Billion [39]. Learning from this outbreak a systematic pandemic readiness and responsiveness plan was developed, suitable for application to any acute respiratory pathogen. The plan’s objective is to “… sustain the nation through the first epidemic wave by minimising mortality and morbidity through the use of measures that are proportional to the assessed public health impact, while ensuring preparedness for vaccination of the entire population when a vaccine becomes available” [40].

The key features of this plan are the establishment of a National Command and Control Structure using the Homefront Crisis Management System (HCMS) hierarchy [41] enabling rapid whole-of-government planning and response, and the establishment of the Disease Outbreak Response System Condition (DORSCON) framework which serves as a centralised planning tool to assist the HCMS to manage the crisis.

Specific components of the pandemic response plan address surveillance, management of suspect cases, border control measures, contact tracing and quarantine, social distancing, communication and social media, and infection control [40]. Within the healthcare system national simulation exercises have been completed to review preparations for emerging epidemics and train staff and administrators in appropriate management processes [42].

Alongside this planning, infrastructure for communicable disease management has been strengthened, and national stockpiles of personal protective equipment (PPE), key medications and vaccines have been established. These preparations have cumulated most recently in the completion of the National Centre for Infectious Diseases (NCID) which was officially opened in August 2019. This is a 330-bed facility co-locating clinical services, public health, research, training, and education [43].

Singapore’s approach to managing this pandemic can be summarised as follows;

1. Establishment of a network of Public Health Preparedness Clinics (PHPCs) which are primary health clinics established in times of national need
2. Hospitalise and isolate the infected
3. Trace contacts rigorously
4. Make social messaging clear

On 31 December 2019, the WHO China Country Office was informed of cases of pneumonia of unknown aetiology detected in Wuhan City, Hubei [44]. At this point Singapore began activating its response plan and implementing non-pharmaceutical measures according to the timeline described in Table 1.

### 1.4. Observed and Expected Economic Impact

In the previous quarter (4Q19), the economy growth was 1.0 % on a year-on-year basis and 0.6% on a quarter-on-quarter seasonally adjusted annualized basis. Generated on advanced evaluations for the first quarter of 2020 (data mainly from January and February 2020), Singapore’s economy has contracted by 2.2% on a year-on-year basis and went down by 10.6% on a quarter-on-quarter seasonally adjusted annualized basis. Taking into account the COVID-19 situation, the Ministry of Trade and Industry (MTI) lowered the gross domestic product (GDP) growth forecast for 2020 to “0.5 to 1.5%” initially in February 2020. In March 2020, the GDP forecast was further reduced to “−4.0 to −1.0%” after considering the unforeseen lower condition of the economy in the first quarter as well as the declining of the external and domestic economic situation since the forecast in February [46]. 

According to Finance Minister Heng Swee Keat, the economic impact of this outbreak will be the severest economic contraction since independence. Singapore’s COVID-19 relief measures up to the end of March add up to 11% of its GDP. The economy still contracted in the first quarter of 2020 with a huge impact on manufacturing, construction, and services despite refraining from a lockdown. This has been the biggest fall since the 2009 financial crisis. Singapore is a main financial hub and port and hence, a key platform for global trade [47]. In addition, Singapore is also a famous tourist destination and has been affected by the virus prevention measures such as travel restrictions. Tourists from China totals up to around 20% of international visitor arrivals. The Singapore Tourism Board (STB) estimated a 25 to 30% decrease in visitor arrivals in 2020 due to the ongoing COVID-19 pandemic [48].

In response to the economic situation arising from the pandemic, the Government announced several fiscal measures; The first package, the Unity Budget (S$6.4 billion), announced in the 2020 Budget on February 18 includes funds to contain the outbreak (S$800 million), the Care and Support Package (S$1.6 billion) to relief households, and the Stabilization and Support Package (S$4.0 billion) to assist businesses. On March 26, a supplementary budget (the Resilience Budget) with further measures amounting to over S$48 billion, including a S$20 billion loan capital, was declared [49]. Another supplementary budget, the Solidarity Budget (S$5.1 billion), was allocated on April 6—the day before the “circuit breaker” distancing measures begin [50]. Due to the extended circuit breaker, the Solidarity Budget has been updated to include additional support including the extension of the Jobs Support Scheme as well as the Foreign Worker Levy Waiver and Rebate until May 2020. Starting 1 May 2020, COVID-19 Support Grant application opens for those who are unemployed due to COVID-19 [51]. Public health measures flatten the epidemic curve but simultaneously steepen the macroeconomic recession curve. However, appropriate macroeconomic measures can flatten the recession curve [52].

### 1.5. Media Coverage 

During the Singapore’s first wave of the COVID-19 pandemic, the Ministry of Health (MOH) Singapore website published updates on the COVID-19 local situation including clarifications on misinformation, total number of imported cases, case summary, and the DORSCON level. Other useful resources found on this website are the infographics and posters such as “Stay Safe with the 5M’s” (manage my health, maintain clean hands, minimize contacts, mask up when unwell and monitor the news). With today’s advanced technology, false information can be easily spread in a short amount of time. There is a need for a real-time information sharing platform which enables authorities to act on the spread of information as well as misinformation on COVID-19 in order to avoid community panic [53]. To curb any misinformation on COVID-19, the MOH reported clarifications on their website. For example, the MOH notified that a fake Singapore General Hospital memo on Prime Minister (PM) Lee Hsien Loong testing positive for COVID-19 has been shared on social media and text messaging platforms [54]. The Government also used social media such as Gov.sg WhatsApp as a platform where the community can sign up for COVID-19 updates, clarifications on misinformation of government policies and key government announcements [55].

In the first few months of the COVID-19 pandemic, Singapore gained international media coverage on their response of the COVID-19 crisis. Prime Minister Lee held several briefings on COVID-19 updates in three languages (English, Malay and Chinese) [56]. The Director General of the WHO, Dr. Tedros Adhanom, praised Singapore more than once. In mid-February 2019, the WHO was amazed at Singapore’s attempt to contain the outbreak by rigorous contact tracing. Dr. Tedros complimented Singapore again in March 2020 at the WHO press briefing; Singapore is a wonderful exemplar of an all-of-government approach including PM Lee’s explanation of the risks and reassurance of the people in his videos [57]. 

Prime Minister Lee has also been interviewed on CNN by Fareed Zakaria on 29 March 2020 regarding Singapore’s response to the pandemic and the impact on Singapore’s economy. In response to people who claimed that Singapore’s paternalistic system enabled them to handle the situation, the Prime Minister said that they have not enforced exceptional powers and are transparent. He also emphasized that people should trust, support, cooperate and have confidence in the government [58].

The stringent measures imposed by the Singaporean Government was also in the news. A resident lost his Singapore permanent residency and will be banned from re-entering Singapore due to breaching the Stay-Home measure [59]. On the other hand, this pandemic has also highlighted the poor living conditions in migrant worker dormitories. Due to several clusters in these dormitories, over 20,000 migrant workers were quarantined. However, the packed housing environment does not allow for social distancing and the unhygienic conditions also promote the spread of the virus [60]. The Manpower Minister, Josephine Teo, announced measures to contain the spread of the disease in the migrant dormitories including moving and testing all essential workers. The workers’ wellbeing is managed in three phases (basic right, medical operations right and recovery right) [61].

### 1.6. Social & Political Disruption

As the community adapted to the various measures implemented, their routines have been impacted. Like other countries, in the initial phase of the pandemic shoppers in Singapore panic bought to stock up on food and household items, especially after the MOH declared DORSCON Orange level, with the concern of shortages. The Government reassured the public that Singapore has sufficient supplies of essential items as well as urged them shop responsibly and that there is no necessity to hoard [62].

Worried about shortages of personal protective equipment (PPE), consumers also scrambled to secure supplies of surgical masks. On the supply side, profiteering of surgical masks by businesses and online platforms taking advantage of the COVID-19 pandemic to increase prices due to the high demand were reported by consumers. The MTI has reminded sellers of their corporate social responsibility [63]. The Government announced that four masks per household has been allocated from its limited stock. In a leaked audio recording of a closed-door meeting with Minister of Trade and Industry Chan Chun Sing and Singapore Chinese Chamber of Commerce and Industry (SCCCI) members, Chan commented that he was humiliated by the panic buying that occurred in Singapore. This chamber’s president pointed out that the leaked recording was a betrayal of trust and this issue will be investigated [64].

Some scammers have also taken advantage of COVID-19 measures implemented. Individuals have been requested for their personal data such as financial information, or collection of documents from the MOH via automated voice calls or scammers impersonating MOH staff or contact tracing team. The MOH has clarified that they do not request for financial information as part of contact tracing [65]. 

Along with the above social issues, mental health also needs to be acknowledged. Some of the measures implemented can have a significant impact of the psychological well-being of humans, especially on at-risk groups. Safe distancing measures such as remote work, distance learning and avoiding social gatherings have caused major changes in daily lives. The unknown characteristics of the virus and absence of an effective vaccine provoke anxiety as the pandemic unfolds. It is crucial that we do not neglect the mental health of frontline health workers. As Chinese Singaporeans are the major ethnicity in Singapore, it is also essential to avoid the stigma against Asians [66]. PM Lee also noted that it is part human nature to be fearful and anxious, but this may be counterproductive. Hence, this period of COVID-19 challenges our solidarity and psychological willpower [67]. 

## 2. Materials and Methods 

### 2.1. Epidemiology

**Figure 1:**
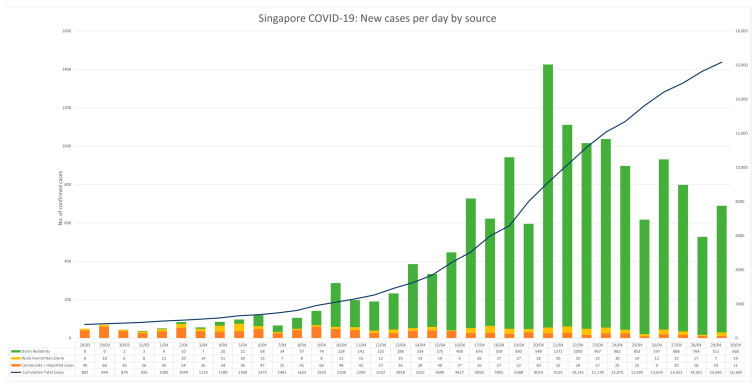
Confirmed cases reported between 27 March–1 May 2020. Source. https://www.moh.gov.sg/covid-19/situation-report. Local Community includes residents, pass holders and work permit holders (WPH) not residing in dormitories.

The following epidemiological data has been retrieved from the Ministry of Health Singapore government website (https://covidsitrep.moh.gov.sg) and their situation reports [68,69]. This data is subject to change due to the rapidly developing situation of COVID-19 in Singapore and globally. As of 1 May 2020, 17,101 cases of COVID-19 were confirmed in Singapore and a further 29,239 persons under quarantine (PUQ) orders, isolating themselves for 14 days at home (*n* = 2058), government facility (*n* = 4296), gazetted dorms (*n* = 4026), non-gazetted dorms (*n* = 15,365) or pending quarantine location (*n* = 3494). Of the first 17,101 confirmed COVID-19 cases, only 3.33% were imported (*n* = 571) whilst 86% (*n* = 14,759) were locally transmitted from migrant workers living in dormitories (Figure 1). 82% (*n* = 14,053) of cases were admitted into care facilities for isolation, 10% (*n* = 1764) admitted to hospitals, 16 deaths and 7.4% (*n* = 1268) cases considered recovered in the first four months.

In Figure 1, Singapore shows a tenfold increase in the number of daily COVID-19 from March–May 2020. This was undoubtedly due to the rapid spread of the virus amongst migrant workers living in dormitories. For this reason, Singapore’s Multi-Ministry Taskforce had more than doubled the number of tests conducted per day from 2900 in early April 2020 to an average of over 8000 (3000 of these conducted on migrant workers living in dormitories) with 2% testing positive [54]. This aggressive testing earned Singapore international admiration for its efforts to contain the spread of the virus. However, exponential growth is still seen as cases continued to increase.

**Figure 2:**
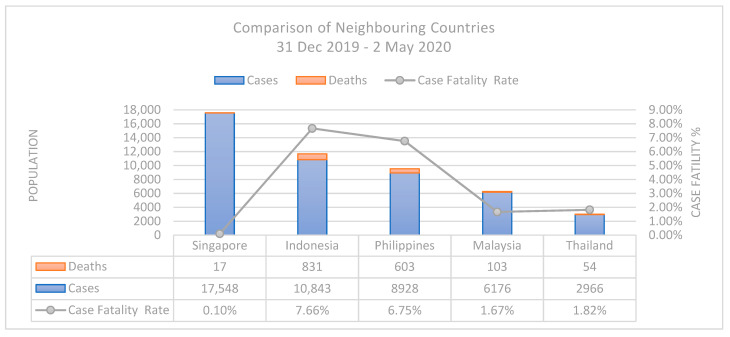
Country Comparison of confirmed cases, deaths and Case Fatality Rate (CFR) reported between 31 December 2019–2 May 2020 [70].

Despite the spike in COVID-19 cases Singapore experienced through March and April, Singapore managed to maintain one of the lowest case fatality rates globally (0.1%). This may be attributed to exceptional health care facilities, hygiene, and mitigation strategies. However, the rise in dormitory cases shifted Singapore’s COVID-19 position, making it one of the ASEAN countries with the most confirmed infections, assuming surrounding countries were reporting cases as thoroughly as Singapore (Figure 2). 

### 2.2. Mathematical Modelling

#### 2.2.1. Doubling Time

From January to April 2020, Singapore experienced two spikes in cases of COVID-19. First, a spike in imported cases during the period 10–27 March, in which imported cases outweighed local cases each day and accounted for 67% of all confirmed cases during that period. The Ministry of Foreign Affairs attributes most of these cases being Singaporean residents returning from overseas travel [71]. Secondly, through the second half of April, there was a spike of local cases amongst migrant workers living in dormitories. 

**Figure A1:**
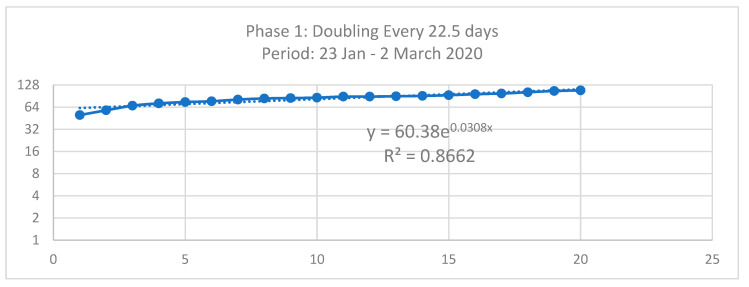
Logarithmic scale. Doubling time calculated based on a 20-day period in Phase 1. Data retrieved from the Singapore Ministry of Health and the WHO [68,69]. Last updated on 1 May 2020.

**Figure A2:**
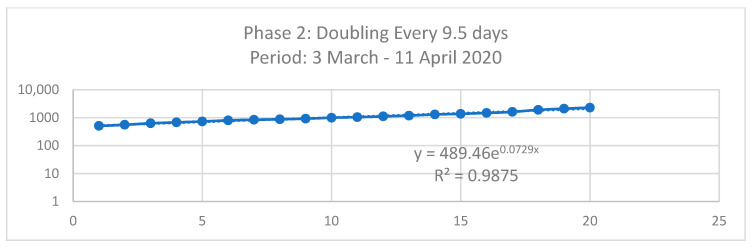
Logarithmic scale. Doubling time calculated based on a 20-day period in Phase 2. Data retrieved from the Singapore Ministry of Health and the WHO [68,69]. Last updated on 1 May 2020.

**Figure A3:**
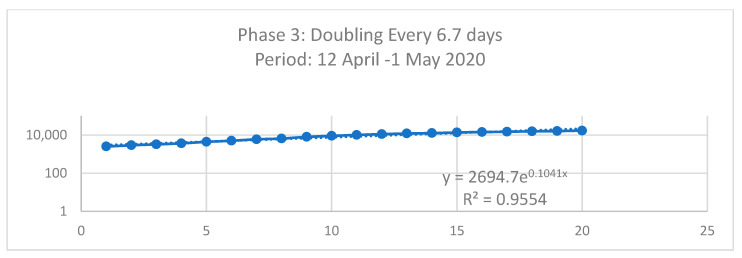
Logarithmic scale. Doubling time calculated based on a 20-day period in Phase 3. Data retrieved from the Singapore Ministry of Health and the WHO [68,69]. Last updated on 1 May 2020.

To model the first 120 days of COVID-19 in Singapore, we first calculated the doubling time. To demonstrate the spikes seen in mid-March 2020 and mid-April 2020, we present doubling times in three phases; Phase 1, first 40 days from 23 January 2020–2 March 2020; Phase 2 will use data from following 40 days 3 March 2020–11 April 2020; and Phase 3 will use data from 12 April–1 May 2020. (Table 2; Appendix A Figure A1, Figure A2 and Figure A3). Using the formula $y=A_{e\cdot }^{Bx}$, we can calculate the doubling time (Td) by the following equation: $\left(Td\right)\;=\;\frac{lnln\;\left(2\right)\;}{B}$.

**Figure A4:**
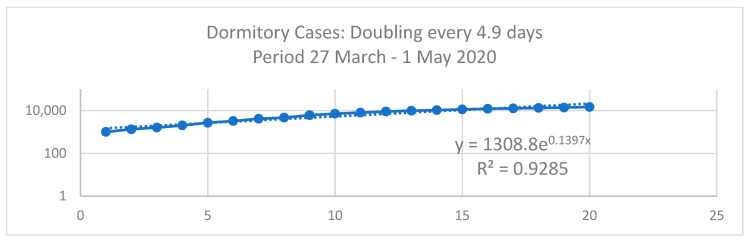
Logarithmic scale. Doubling time of conformed Dormitory cases in Singapore. Data retrieved from the Singapore Ministry of Health and the WHO [68,69]. Last updated on 1 May 2020.

**Figure A5:**
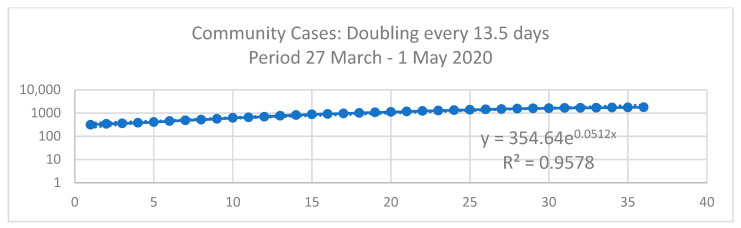
Logarithmic scale. Doubling time of conformed Community cases in Singapore. Data retrieved from the Singapore Ministry of Health and the WHO [68,69]. Last updated on 1 May 2020.

As stated previously, we can attribute the influx of Singaporeans returning from overseas in mid-March 2020 to be the reason for this dramatic change in doubling time from Phase 1 to Phase 2; and the outbreak of COVID-19 in migrant workers living in dormitories to be the cause of the further increase in speed of doubling time in Phase 3. To compare the effect housing can have on an outbreak, we compare the doubling times cases of those living in dormitories and cases of the rest of the local community (Appendix A Figure A4 and Figure A5). From this data, we can infer the doubling time of COVID-19 cases in dormitories is almost three times the rate as cases within the rest of the community. This is a major concern for Singapore with such a large population of migrant workers living in dormitories [22]. 

#### 2.2.2. SEIR Model

To demonstrate the prediction of the COVID-19 pandemic in Singapore during the months Jan–May 2020, we used the *Susceptible-Exposed-Infected-Recovered* (SEIR) epidemic model following the guidelines presented by the Institute of Disease Modelling [7]. The models presented reflect Singaporean residents residing in dormitories as well as predictions for the local community not residing in dormitories. To proceed, we make a few assumptions; the entire population of Singapore is ‘susceptible’ to the disease at the same rate and the population remains constant. This model does not consider the health of each individual nor their age. This population is then divided into four categories in relation to contracting the disease at time(*t*); those susceptible (*S_t_*); those exposed (*E_t_*); infected (*I_t_*); and those removed/recovered (*R_t_*) namely those who have recovered from the disease and confer immunity or those who have died. 

The SEIR Model uses *time (t)*, measured in days, as an independent variable and three dependent variables. Firstly, persons in each SEIR category as a function of time, denoted as follows.

S = S(t); is the number of susceptible individuals at time t,
E = E(t); is the number of exposed individuals at time t,
I = I(t); is the number of infectious individuals at time t, and
R = R(t); is the number of recovered/removed individuals at time t.

Secondly, the fraction of the total population (*N*) in each category. The mid-year 2019 estimated population of Singapore given by the department of statistics Singapore is 5,703,600 (*n* = 5,704,000) however we will split this population into Dormitory (*n* = 323,000) and Community (*n* = 5,381,000) [64] and we note S+E+I+R=N. 

To determine the rate of infection (β), we considered the incubation rate (*σ*), recovery rate (*γ*), and the basic reproduction number (*R*0) [72]. Initial studies estimated *R*0 for COVID-19 to be between 1.4–3.8 with a median value of 2.2, denoting on average each infected person infects 2.2 others [73]. In order to estimate the initial basic reproduction number for dormitory cases and community cases, we will trial values of *R*0 from the suggested estimated range (1.4–3.8) to demonstrate various predictions of the COVID-19 pandemic in Singapore for migrant workers living in dormitories and the rest of the population not confined to these spaces. 

Several studies have suggested the incubation period (*Y*) of COVID-19 can range from 4–8 days with a median incubation period of 5.2 days [74,75,76]. We will use this median for our study and derive the incubation rate (*σ*) using the following formula [76]

$$\sigma =\frac{1}{Y};\;therefore\;\sigma =\frac{1}{5.2}=0.192$$

Using data from Singapore’s Ministry of Health, it took 13 days for Singapore’s first recovery from COVID-19 which seems consistent with previous research suggestions of an average duration of 14 days [77]. We used this average of 14 days for our study and derived the rate of recovery (*γ*) using the following equations where *D* is the average duration of recovery calculated by total duration (14 days) incubation period (5.2 days) [72]

$$D=\frac{1}{\gamma };\;\mathrm{therefore}\;\gamma =\frac{1}{D}=\frac{1}{8\cdot 8}=0.1136$$

As we do not know the rate of infection (*β*), we use the following equation [72].

$$R0=\frac{\beta }{\gamma };\;therefore\;\beta =R0\;x\;\gamma$$

Table 3 gives the equations [7] and values required to predict the COVID-19 pandemic for Singapore using the SEIR model. Through contact tracing, the Ministry of Health Singapore has identified 28,140 close contacts who have been quarantined for 14 days. Of these, 16,052 have completed their quarantine. We can therefore determine the estimated initial number of Exposed individuals (*E*) by diving those who have completed their quarantine by the number of confirmed cases 14 days prior. Dividing the suspected cases (*n* = 16052) by the confirmed cases (*n* = 879) will give us the approximate initial number of Exposed individuals (*n* = 18). 

## 3. Results 

Using the SEIR model, we can determine the basic reproductive number of actual COVID-19 cases in Singapore with dormitories and the local community. 

**Figure 3:**
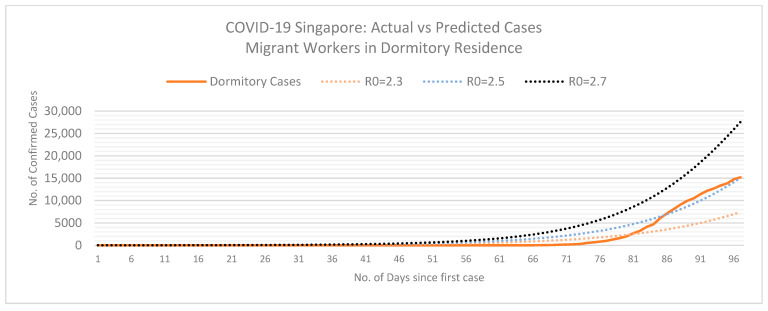
Exponential growth of COVID-19 in Singapore of Migrant Workers living in Dormitories. Dotted lines represent differing COVID-19 basic reproduction numbers (*R*0). Data retrieved from the Singapore Ministry of Health and the WHO [68,69]. Last updated on 2 May 2020.

**Figure 4:**
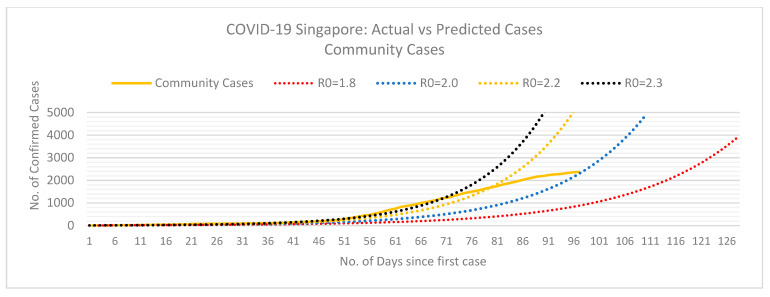
Trajectory of COVID-19 in Singapore of community cases including permanent residents, pass holders and work permit holders (WPH) not residing in dormitories. Dotted lines represent differing COVID-19 basic reproduction numbers (*R*0). Data retrieved from the Singapore Ministry of Health and the WHO [68,69].

From Figure 3 and Figure 4, we can assume the basic reproduction number (*R*0) for Singapore’s dormitory cases is approximately 2.5. However, at this rate, Singapore must review their mitigation measures in dormitories to reduce the rate of infection amongst their migrant population and prevent a burden on their health care system. The exponential curve indicates a possibility that *R*0 will increase if the measures implemented do not contain the spread of infection in dormitories. On the contrary, the basic reproductive number for community cases seems to be decreasing and stands at 2 at the time of this study. As of May 2020, Singapore seems to be flattening the curve for community cases.

**Figure 5:**
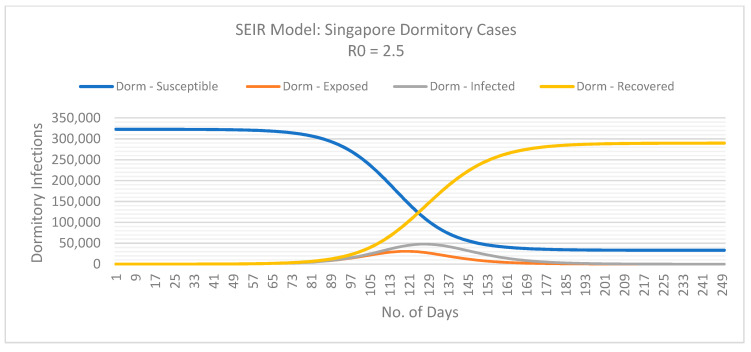
Predicted dynamics of the COVID-19 outbreak within the migrant worker population in Singapore living in dormitories. Basic reproduction number (*R*0) is 2.5. Data retrieved from the Singapore Ministry of Health and the WHO [68,69].

**Figure 6:**
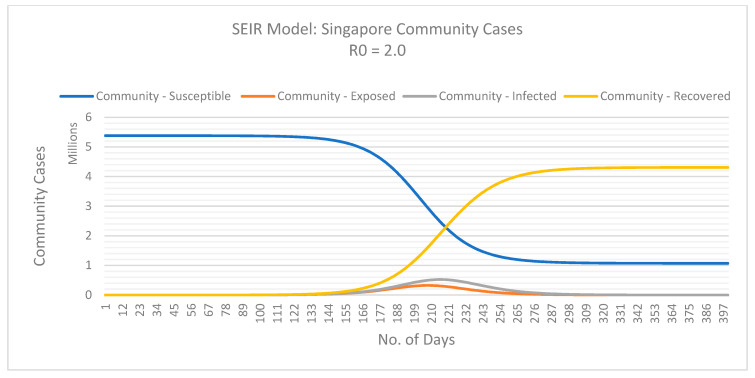
Predicted dynamics of the COVID-19 outbreak within Singapore’s local community not living in dormitories. Basic reproduction numbers (*R*0) is 2.0. Data retrieved from the Singapore Ministry of Health and the WHO [68,69]. Last updated on 2 May 2020.

The following SEIR model graphs provide predictions for dormitory (Figure 5) and community (Figure 6) cases if these basic reproduction numbers persist. 

## 4. Discussion

In the first three months of COVID-19 pandemic, Singapore demonstrated exceptional containment of the spread of the virus however, our results show an exponential growth in the number of infections from April 2020. Singapore experienced an almost tenfold increase in the number of new cases per day in April, however, despite this increase, Singapore remained one of the countries with the lowest case fatality rate (0.1%). As of early May 2020, infections within Singapore’s migrant workers living in dormitories were doubling every 4.9 days, this was almost triple the doubling rate of infectious amongst the rest of the population. Our SEIR model indicated Singapore’s basic reproductive number (*R*0) for those residing in dormitories was approximately 2.5 and a lower reproductive number of 2.0 for the rest of Singapore’s population. This implies an infected person in a dormitory will on average infect 2.5 other susceptible people however someone not living in these dormitories will infect 2 susceptible people on average. Although the simulations predicted in this case study are based on a homogeneous population, they nevertheless provide insight into the spread of the virus in various living conditions. By analysing the various sub-group populations within Singapore, our results attributed this exponential growth to migrant workers living in dormitories. Such living condition can make implementing mitigation strategies such as self-isolation and physical distancing difficult.

Comparative studies have shown that countries that adopt strategies of prompt lockdown combined with rigorous testing, prompt isolation, and strict enforcement of physical distancing are successful at containment of local COVID-19 transmission [78]. Additionally, lockdown to contain COVID-19 is a window of opportunity to prevent subsequent local epidemic waves [79]. Consideration to various living conditions in Singapore is needed when revising mitigation and exit strategies. Although self-isolating is recommended, implementation cannot be guaranteed, especially for those living in shared accommodation. A study of compliance of home-based isolation showed over 50% of new infections could be averted if self-isolation had taken place in dedicated facilities for mild to moderate COVID-19 cases [80]. Additionally, reviewing social and geospatial networks of migrant workers and locating essential services (such as grocery stores and collection points for remittance) close to migrant worker dormitories can successfully minimise excess gatherings at these potential hotspots [81].

If Singapore had maintained its initial trajectory, without adapting their mitigation strategies, our model predicted the peak (maximum) infectious to reach almost 50,000 (*n* = 47,891) migrant workers living in dormitories by 1 June 2020 (day 127). As for the remaining community, by 1 June 2020, Singapore was expected to have an additional 13,000 infections (*n* = 12,922). If Singapore reduced its reproductive number to at least 2.3 for those living in dormitories, this will significantly reduce the number of infections by almost 13,500 by 1 June 2020 (*n* = 34,401), ultimately reducing the burden on their health care systems and possibly further economic and societal disruptions. 

While our results estimate the progression of COVID-19 on Singapore’s sub populations, our model is however limited. To enhance these predictions with authentic outcomes, consideration needs to be given to the heterogeneity Singapore’s population, the various mitigation measures taken and Singapore’s migration patterns. The Institute for Disease Modelling [7] suggests employing the Epidemiological MODeling software (EMOD) to simulate complex dynamics underlying disease transmission. Such a model will be able to incorporate Singapore’s migration patterns, climate, various mitigation interventions and heterogeneity in its population demographics.

Due to its location and status as a busy transport hub, Singapore is vulnerable to communicable diseases. Having already been exposed to past pandemics such as the SARS outbreak in 2003 and H1N1 in 2009, Singapore was well-prepared for the COVID-19 pandemic. Singapore’s proactive response in implementing mitigation measures such as contact tracing and surveillance in the early stages of the pandemic along with compliance with the general public most likely played a significant role in containing the virus in the early months, leading to very low rates of general community transmission by the end of April 2020. Transmission rates amongst the migrant worker population however tell a different story. The rapid growth in cases particularly in the large dormitories highlight the significant challenges Singapore must overcome in addressing this vulnerable population. Emergency measures are underway to deploy resources to this community and to rapidly test workers and attempt to isolate new cases by relocating them to temporary accommodations [82] and Singapore’s response capabilities will be significantly tested.

While other island nations like Australia have been able to minimise the importation of COVID-19 through strict border controls including complete bans on international travel [83], Singapore’s dependence on the free movement of trade and people has required a more nuanced response, and if local disease transmission is not rapidly brought under control the long-term damage the COVID-19 pandemic will certainly be significant. Addressing the living conditions of Singapore’s migrant worker population is also key to containment of the COVID-19 outbreak. Mitigation measures will need to address the hygiene practices available in such high-density housing environments which do not allow for social distancing. Additionally, addressing issues of mental health and social security will be equally as crucial.

## 5. Conclusions

Although studies have shown these mitigation strategies to be effective, careful consideration also need to be given to the social, political and economic impacts for those living in vulnerable conditions. Isolation and lockdowns can have negative social consequences such as impacts on mental health, education, food security and access to health care especially for those living in socially and economically vulnerable conditions [84].

It is difficult to predict how much longer the country (and the rest of the world) will need to implement stringent mitigation measures and containment of the COVID-19 pandemic. Given Singapore’s wealth, high life expectancy, effective communication strategies and community support, Singapore will most likely recover well from the COVID-19 pandemic however the message to all countries from the Singapore experience to date is to ensure that pandemic preventative measures are broadly and robustly implemented across the complete society, not just citizens and permanent residents. 

## Figures and Tables

**Table 1:** Timeline of non-pharmaceutical measures implemented by Singapore in response to the COVID-19 pandemic [45].

Date of Implementation	Measure(s)
2-Jan	Singapore MOH issues health advisory and temperature checks at Changi Airport from Wuhan arrivals initiated
20-Jan	Temperature screening at Changi Airport was extended to all travellers coming from China. The public advised to adopt good personal hygiene measures and seek medical attention promptly if they are unwell.
23-Jan	First confirmed local case, border control measures enhanced and extended to land and sea checkpoints. First meeting of whole-of-government response taskforce
25-Jan	MOH imposed a visitor limit of two per patient in hospitals to slow the spread of the virus. Some hospitals have discouraged children from visiting.
31-Jan	New visitors with travel history in mainland China or with PRC passports are not allowed entry
1-Feb	Surgical masks issued to each household to alleviate shortages and price gouging. Only people who are unwell should wear masks. Safe distancing measures progressively implemented.
3-Feb	Precautionary Measures advised in Preschools including travel declarations, temperature screening and health checks
7-Feb	Risk assessment raised to DORSCON Orange Contact tracing of all confirmed cases Quarantine of all recent travellers to Hubei Activation of Public Health Preparedness Clinics (PHPC)
14-Feb	PHPC system is in place, establishing 900 GP clinics specially designated to manage people suffering from respiratory illness
17-Feb	Implementation of 14 day stay-home notices for all travellers returning from mainland China
25-Feb	Travel restrictions implemented for visitors from some areas of the Republic of Korea
3-Mar	Travel restrictions implemented for visitors from Iran, Northern Italy, Japan and the Republic of Korea
13-Mar	Travel restrictions implemented for visitors from Italy, France, Spain and Germany, as well as additional social distancing measures within Singapore.
17-Mar	Singaporean students studying overseas advised to return home
18-Mar	Singaporeans advised to defer all travel abroad. All inbound visitors must remain in their place of residence for 14 days
21-Mar	Government Technology Agency launches the TraceTogether application which can use Bluetooth technology to track close contacts, enabling wide scale contact tracing
23-Mar	Travel restrictions all short-term visitors are not allowed to enter or transit through Singapore. Returning Citizens and residents are issued 14 day stay at home notices
24-Mar	Tighter measures to minimise further spread of COVID-19 implemented including closing all bars and entertainment venues. Schools and retail malls remain open.
26-Mar	All gatherings outside of school and work limited to 10 or fewer until 30/4
27-Mar	Ministry of Education announces 1 day of Home-Based Learning (HBL)
7-Apr	‘Circuit Breaker’ measures implemented 7 April to 4 May All business, social, other activities that cannot be conducted through telecommuting suspended Essential services (supermarkets, delivery services) and entities part of the global supply chain remain open All schools shifted to HBL
18-Apr	180,000 foreign workers in the construction industry issued stay-home notices
21-Apr	‘Circuit Breaker’ measures extended for 4 weeks through to 1 June 2020
1-May	An inter-agency taskforce of 3000 officers from six public agencies including the Ministries of Manpower and Health, and the Singapore Armed Forces is established. This taskforce will: Investigate all aspects of workers well-being Establish onsite medical facilities at all 43 purpose-built dormitories and medical posts at 760 foreign workers’ recreation centres Deploy 12 mobile medical teams with up to 50 personnel to provide field support Provide safe transport and shuttle services to enable workers to visit medical posts.

**Table 2:** Doubling times of COVID-19 progression in Singapore from 23 January–1 May 2020.

y = Ae^Bx^	Phase 1	Phase 2	Phase 3	Dormitory	Community
Doubling Time (Td) = Ln(2)/B	0.0308	0.0729	0.1041	0.1397	0.0512
	22.5 Days	9.5 Days	6.7 Days	4.9 Days	13.5 Days

**Table 3:** Equations and values for SEIR Model. Raw data for the SEIR Model was retrieved from the Singapore Ministry of Health COVID-19: Cases in Singapore. Last updated 2 May 2020.

Equations	Values	Definitions
*R*0 = *ß*/*ƴ*		*S* = Susceptible; initial
*β*: rate of spread; *β* = *R*0*γ		*E* = Exposed; initial
*Y*: duration of incubation	5.2 days	*I* = Infected; initial
*σ* = 1/*Y*; rate of latent (exposed)individuals becoming infectious	0.192	*R* = Recovered; initial
*D*: average duration of recovery = total duration incubation period	8.8	*ß* = Rate of spread of infection
*γ*: Recovery rate; *γ* = 1/*D*	0.113	*σ* = Incubation rate
*Sn* = *Sn* − 1 ((*Sn* − 1/*S*) * (ß*In − 1))		ƴ = Recovery rate
*En* = *En* − 1 + (Sn − 1/*S*) * (ß*In − 1) (*En* − 1*σ)		*T* = time interval; usually days
*In* = *In* − 1 + (*En* − 1*σ) (*In* − 1*ƴ)		*n* = number of people on day *n*
*Rn* = *Rn* − 1 + (*In* − 1 * *ƴ*)		*N* = total number of people = *S* + *E* + *I* + *R*

## Data Availability

Data used to support the findings of this case study are publicly available at https://www.moh.gov.sg/covid-19/situation-report, https://www.who.int/emergencies/diseases/novel-coronavirus-2019/situation-reports, and https://qap.ecdc.europa.eu/public/extensions/COVID-19/COVID-19.html.
